# Meeting People's Needs in a Fully Interoperable Domotic Environment

**DOI:** 10.3390/s120606802

**Published:** 2012-05-25

**Authors:** Vittorio Miori, Dario Russo, Cesare Concordia

**Affiliations:** Institute of Information Science and Technologies “A. Faedo” (ISTI), CNR-National Research Council of Italy, Via G. Moruzzi 1, Pisa 56124, Italy; E-Mails: dario.russo@isti.cnr.it (D.R.); cesare.concordia@isti.cnr.it (C.C.)

**Keywords:** Ambient Intelligent, domotics, home environment, interoperability, DomoNet, web services, XML, data mining, machine learning, association rules

## Abstract

The key idea underlying many Ambient Intelligence (AmI) projects and applications is context awareness, which is based mainly on their capacity to identify users and their locations. The actual computing capacity should remain in the background, in the periphery of our awareness, and should only move to the center if and when necessary. Computing thus becomes ‘invisible’, as it is embedded in the environment and everyday objects. The research project described herein aims to realize an Ambient Intelligence-based environment able to improve users' quality of life by learning their habits and anticipating their needs. This environment is part of an adaptive, context-aware framework designed to make today's incompatible heterogeneous domotic systems fully interoperable, not only for connecting sensors and actuators, but for providing comprehensive connections of devices to users. The solution is a middleware architecture based on open and widely recognized standards capable of abstracting the peculiarities of underlying heterogeneous technologies and enabling them to co-exist and interwork, without however eliminating their differences. At the highest level of this infrastructure, the Ambient Intelligence framework, integrated with the domotic sensors, can enable the system to recognize any unusual or dangerous situations and anticipate health problems or special user needs in a technological living environment, such as a house or a public space.

## 1. Introduction

The Ambient Intelligence (AmI) vision described by Weiser [1] is still far from becoming a reality, though many steps forward have been taken over the last few years. Research activities are continuously proposing new services and algorithms able to provide ever more powerful and sophisticated solutions. The Ubiquitous Computing, Ambient Intelligence [2], and Context-Aware paradigms paint a picture of future society as one in which humans will be surrounded everywhere and at all times by intelligent interfaces embedded in everyday objects, such as furniture, clothes, vehicles, roads and smart materials. This vision foresees high-capacity connectivity, by which people and objects are able to interact with each other and with the environment. One prerequisite for realizing such a vision is that the environment, in turn, be able to identify and locate users. Moreover, the technological infrastructure should remain in the background, in the periphery of our awareness, and should only move to the center if and when necessary. Computing thus becomes ‘invisible’.

In such vision, the home of the future will surely be a more technologically rich environment, offering tens or even hundreds of pervasive network-based services ranging from simple video entertainment streams to complex home energy-management packages. Some of these services will be provided by physical appliances within the home and others by external service providers.

Such services enable anyone to operate a wide range of domestic appliances and control other vital home functions. In recent years attempts to enhance the autonomy of the elderly, the sick and the disabled have begun to exploit easily available technologies. Environmental controls can be considered effective aids to enhance many users' functional capabilities. People with disabilities may be completely unable to physically manipulate objects in their environment. Thus, a domotic environment which may provide simply useful services to most, can, to a disabled user, become a necessity for overcoming personal physical limitations. The environmental system may be the only way for such people to control the world around them. The typical items within the home that can be controlled are: lighting, windows and blinds, doors, loudspeakers, heating, ventilation, air-conditioning, home appliances (refrigerator, washing machine, cooker, *etc.*), audio-video equipment, burglar and fire alarms, telecommunications.

Many operations control sequences could be automated to save the user the effort of adjusting the system themselves. Thus, a great improvement to such control would come from adding a degree of “intelligence” to the system by enabling it to recognize recurrent activities, unusual or dangerous situations in order to anticipate health problems or special home user needs. Such problems may be addressed by monitoring users' habitual activities, which enables creating rules-based profiles in order to capture and formalize their normal behavior. However, many people have privacy concerns about having their activities monitored, the rules for monitoring implementation and what information is transmitted, and to whom. Such concerns must obviously be addressed through appropriate privacy agreements and safeguards.

One existing solution for modeling the complex interactions occurring between independent but interoperable entities within an intelligent environment is to implement the so-called Intelligent Agent paradigm [3], whose features make it particularly appropriate for intelligent management of autonomous, proactive devices and entities able to communicate, interact and coordinate with each other [4].

Given the heterogeneity of these services, it will be up to suitable support middleware to make these services discoverable by and accessible to residential environments. Although some domotic and smart home technologies are currently operational and ready to realize such scenarios, they have as yet not been able to garner a broad consumer market, some of the main causes being the lack of standards and interoperability, as well as the absence of any “must-have” new functionality provided at an appealing cost.

Focusing on the residential environment, Ambient Intelligence may be seen as the layer on top of the domotics, *per se*. Its aim is to progress from the mere programming of isolated devices, to integrating them in order to achieve global, unified goals. Home networks, that is, the specific Local Area Networks for home environments, include both networks to the home (access networks) and networks throughout the home.

Following this vision, the domestic network must connect all household appliances and interact with the personal area network and the ‘body network’ of each person in the home. Within homes, local networks with potentially different underlying implementations can be combined, or at least managed, as one logical network. The need to support interoperability services such as bridges, gateways or adapters between different networks in the backbone, as well as in access and local networks is an essential prerequisite for all AmI applications.

The emerging field of domotics as yet suffers from a number of shortcomings, first amongst which is a lack of definition of application requirements. It has thus far offered a large number of only *ad hoc* solutions, which unfortunately are often limited in scope and difficult to integrate. In order to make possible the advent of genuine AmI applications, it is crucial that standard interoperability protocols be defined and implemented.

## 2. Related Works

In recent years several interesting and innovative solutions have emerged regarding interoperability and Ambient Intelligence. Numerous proposals have moreover been advanced to enable future smart houses to adapt to user needs.

Regarding interoperability and systems integration, Twente University [5] has proposed a “cluster cultures” approach that aims to support heterogeneous technologies (including legacy ones). Using a “touch and play” system at device registration time, the system provides a zero-configuration environment for the exchange of credentials among its gateways and registers device services in a hierarchical structure. The architecture provides a high level of security by using cryptographic algorithms. The same goal has been pursued at Waseda University [6] which has proposed a framework designed to easily integrate legacy middleware, services and clients by exploiting a Virtual Service Gateway. This connects one piece of middleware to another by exploiting a Protocol Conversion Manager, whose task is to convert the different middleware protocols into the specific internal protocol used by the Virtual Service Gateway. Information about the location and functions of services are provided by a Virtual Service Repository. The Building Information Exchange Group (OBIX) is developing a comprehensive set of standards using XML and Web Services for facilitating home as well as building environment information exchanges between heterogeneous systems [7]. Another interesting project is the “Domotic House Gateway” [8]. It implements an event-based mechanism which is used to exchange messages between the single device and the system. Such events are then converted internally into logical events so as to clearly separate the actual physical issues from the semantics that goes beyond the devices and their role within the house. One level of the architecture implements a rule-based core that can be dynamically adapted either by the system itself or manually through external interfaces. Each device requires a device driver, which is responsible for translating its low level or hardware states and activities into events that can be managed by the system. Lastly, Amigo [9] is an important European project aimed at studying and developing Ambient Intelligence features for the networked home environment and improving the usability of such systems. Its main goals include three major guidelines: user-friendly interfaces, interoperability and automatic discovery of devices and services.

In general, the most common approach to learning user habits is to monitor their behavior via rules that analyze how appliances and services interact with each other and the user. Chin [10] has proposed a taxonomy that classifies the creation of system rules using three criteria: *pre-programmed*, whereby the rules are usually created by the developers or manufacturers; *user-programmed*, when the rules are created through end-user programming; and *agent-programmed*, when the rules are created by intelligent agents, artificial intelligence or machine learning mechanisms.

Adopting *user-programmed* rules leaves the task of configuring the system up to the end user, as in the Humble project [11], in which users build the system through puzzle-like graphical programming representations, the CAMP project [12], which adopted a pseudo-natural language interface to enable non-technical people to create context-aware ubiquitous applications, and Rivera-Illingworth *et al.* [13], who propose a system that uses a temporal neural network-based agent to recognize behaviors such as listening to music, working at the computer or sleeping, by using a special user interface in order to label the various user activities. Unfortunately, these kinds of systems tend to be very complex and therefore difficult for end users to configure properly.

We instead believe it preferable that no voluntary actions be required of users for configuring the intelligence system. Our approach, however, uses no pre-fixed rules, and thereby offers the advantage of offering fully personalized, flexible system services following the *agent-programmed* approach.

Since our system does not initially possess any information regarding users' habits and no tagged training examples are used, the goal of finding relations and groupings between similar data has been achieved using an unsupervised machine learning algorithm to capture and record such activities in the system via continuous monitoring of the interactions between users and the domotic devices.

An *agent-programmed* approach has also been pursued by MavHome [14], which applies prediction algorithms to forecast future user actions, as well as the TIAX company, who, together with the Massachusetts Institute of Technology (MIT), has set up PlaceLab [15]—a living laboratory for studying human behavior, routine user activities and the interactions of everyday life.

In this context, Tapia [16] has applied the *Navie Bayesian classifier* to recognize everyday life activities, such as bathing, toileting, dressing and preparing lunch, by analyzing data collected from a set of simple small state-change sensors. In recent years, data mining techniques have been used to process data captured from a collection of sensors placed throughout the environment. Lühr [17], having arrived at conclusions similar to our own, uses the data mining method known as *association rule learning* to detect new behaviors occurring within smart homes. Finally, Zheng [18] uses a self-adaptive neural network called Growing Self Organizing Maps to execute cluster analysis of day-to-day human activities.

Numerous contributions have also proposed various other methods of analyzing human activity. Aggarwal [19] describes how complex human activities can be recognized through the use of video sources. In particular, he provides a detailed overview of various state-of-the-art research papers on human activity recognition for surveillance purposes. He discusses both the methodologies developed for simple human actions and those for high-level activities. A taxonomy-based approach is followed, and the advantages and limitations of each approach compared. BB6-HM [20] is a system that has been applied to build classifiers for the detection of basic events and visual attributes using heuristic rules and machine learning techniques. It enables recognizing the various parts of the human body as depicted in videos and is used especially in surveillance applications. AmIHomCare is another research project aiming to achieve accurate recognition of daily activities using an agent-based architecture within an intelligent environment [21]. Each agent in the system is responsible for managing a different aspect of the process of recognizing and composing the user's actions. The actual recognition of user activities is based on ontologies and stochastic context-free grammar to determine the setting within the house. Atallah [22] has focused on the use of wearable sensory accelerometers. He has developed a system able to indicate the ideal placement of such sensors on the body in order to best recognize and identify predetermined daily activities.

Other noteworthy work on the use of agents has been published by Isern [3], who describes 15 systems for health care applications, and GerAmi [23], who illustrates a distributed AmI system, built using intelligent agents, to support Alzheimer patients and the elderly. This solution allows for locating people through techniques based on RFID sensors, the collection of historical patient data and the management of alerts to be sent to physicians and paramedics through special purpose PDAs.

Xefteris [24] has proposed a system for identifying and recognizing a user's physical and psychological state through the use of non-intrusive biometric sensors together with pattern- and activity-recognition techniques. The work's aims include building a classification of emotions, detecting movement and identifying individuals suffering from various diseases in different stages who may need supervision in their daily activities. Xefteris also analyzes the proposed solutions in terms of the ethical and legal ramifications of each.

### 2.1. Evolution of Home Automation Systems

In early domotic systems, the component devices were mainly independent of each other, or sometimes grouped into small independent subsystems. Essentially, the hardware and the software were tightly linked and any changes in one system component would likely require updates to the whole system.

The two main challenges facing designers and developers of such early domotic systems were to: (i) physically connect devices and subsystems and (ii) achieve fully modular software systems. Physical connection of domotics devices is an important issue, one major challenge being to build an efficient network while reducing as much as possible the number of devices and cables that have to be added to an existing home or building.

In order to achieve a working domotic system, all the sensors and actuators must be able to ‘talk’ to each other. Moreover, continuous information exchange must be maintained between the sensors and the system, and any action (opening a door or turning on lights) must be initiated by sensor data, rather than merely having a electric charge activate them.

A number of standard protocols have been created in recent years to enable communication between network devices; a list is shown in Table 1. One major trend in today's domotic systems is the adoption of wireless communication technologies [25].

However, domotic communication standards vary greatly one from the other and in most cases are designed for specific purposes. For example, *ZigBee* and *Z-Wave* are suitable for the sensor systems monitoring a person's health (measuring heart rate, pressure, *etc.*), but they do not support management of audio and video, as do *HAVI* or *UPnP* (Table 1).

**Figure 1.**
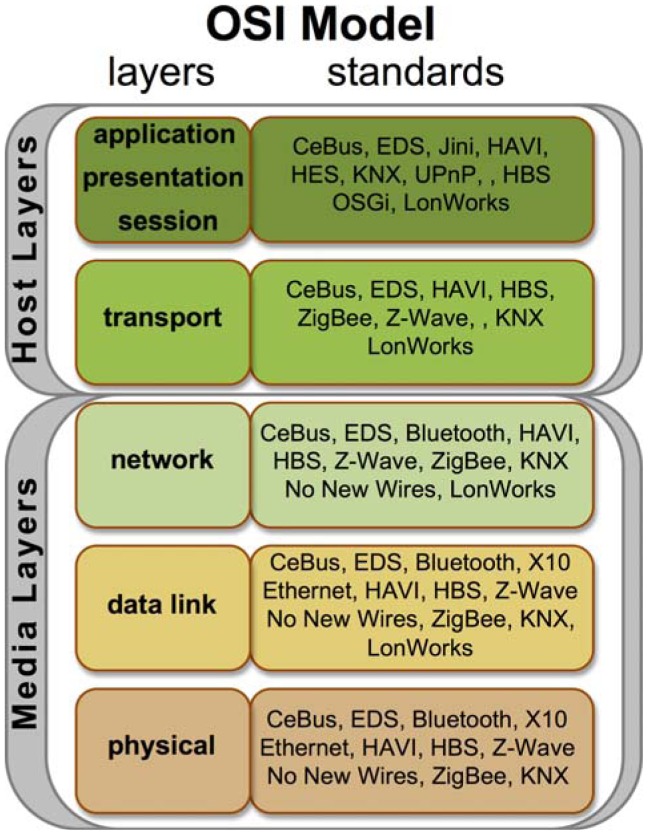
Classification of domotic standard according to the ISO/OSI architecture.

Moreover, some standards have been proposed with the intention of creating home networks, others with the sole purpose of managing them, so existing domotic network protocols can be classified according to the *ISO/OSI* model, which divides systems components according to the particular networking level at which they operate (physical, data link, network, transport and application). Thus, the *KNX* standard, which covers all network levels, is present in each level, while low-level standards, such as Bluetooth, are to the contrary found only in the lower levels. An example of such a breakdown is shown in Figure 1.

Today's most advanced domotic systems have been implemented using platforms whose functionalities are provided as ‘services’, and the interactions between these services occurs via ‘messages’. The *DomoNet* framework, discussed in details in the next section, has been designed following this approach.

## 3. The Interoperability Framework

The many domotic systems that are crowding today's market are rarely interoperable and thus do not permit consumers to choose devices according to their requirements or other relevant criteria, such as cost, performance, trends and confidence, without having to worry about issues of compatibility with their existing systems. Unfortunately, current market practice generally binds consumers to proprietary technologies, forcing them to purchase only devices that conform to a specific system in order to obtain a suitable level of interoperability.

Examples of current, but incompatible domotic protocols are *X10*, *KNX*, *LonWorks*, *UPnP*, *HAVi*, *Jini*, *etc.*, which support various different communication standards (*Ethernet*, *FireWire*, *Bluetooth*, *ZigBee*, *IrDA*, *proprietary buses*, *etc.*). However, at present no domotic technology has the potential to play a leading role in standardizing the field.

To achieve significant results in true interoperability, a middleware infrastructure offering a high level of abstraction is thus necessary. A comprehensive approach is required, rather than addressing technology mismatches directly through the use of *ad hoc* mappings of different specific standards.

By focusing on functionalities and standard solutions, we must first identify a suitable way to describe and define a coherent process to control a variety of devices using well-proven, standard technologies. The most suitable choices appear to be *XML-based* representation for description, and *Web Services* for control.

The interoperability issue has been tackled by our laboratory by creating a digital ‘ecosystem’ called *DomoNet* [26], an open source software released under the *GPL* license, written using the *Java* language and open source libraries and tools. The core of *DomoNet* consists of middleware based on *Web Services* and *Service Oriented Architecture (SOA)* [27] paradigms, in which the services coincide with the functionalities offered by the devices in question.

**Figure 2.**
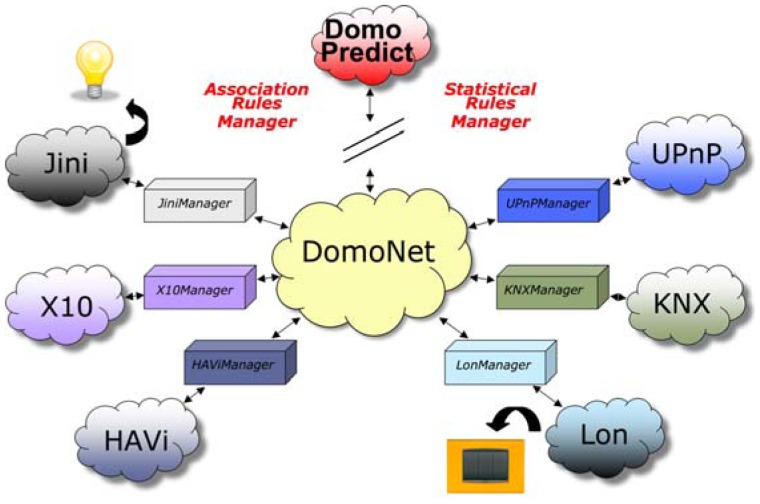
The DomoNet framework.

At the center of the *DomoNet* framework is *domoNetWS*—a *WebServices-based* engine whose task is to enable true cooperation between nodes. It constructs a unique view of the system, including all the devices belonging to all the different domotic systems available through a set of modules that work as gateways, called *TechManagers*, to deal with specific domotic systems. Figure 2 shows a schematic representation of *DomoNet*, showing, by way of example, a switch operated through *LonWorks* technology controlling a lamp managed by the *Jini* domotic system.

*DomoNet* defines a standard language, called *DomoML*, for the semantic abstraction of heterogeneous systems in order to describe device functionalities, data types, messages and models of the interactions and communications among framework entities. It represents a sort of universal domotic language to be applied in any domotic context.

**Figure 3.**
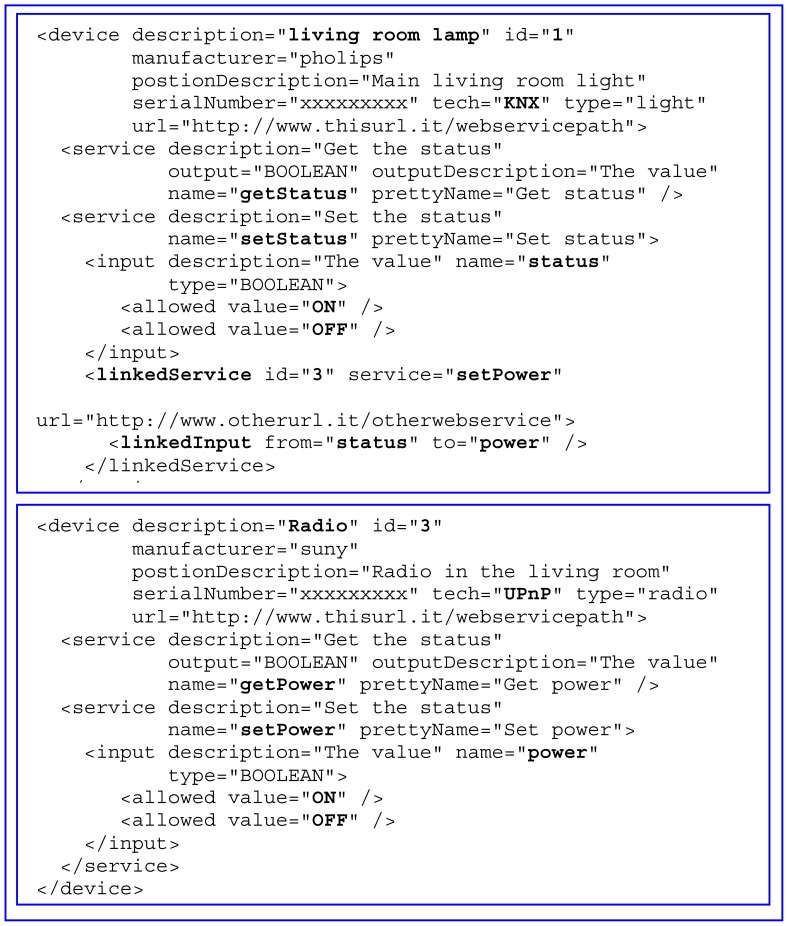
Examples of domoDevice.

**Figure 4.**
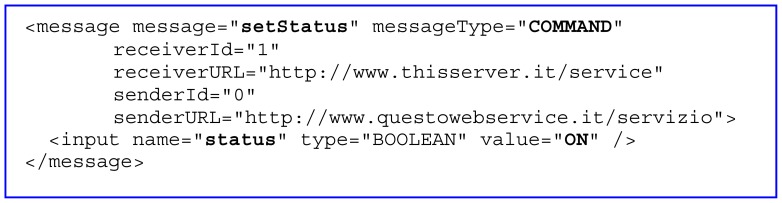
DomoMessage example.

*DomoML* consists of two main formalisms:

- *domoDevice*: provides a formalism to define devices and their functionalities. In particular, it describes the characteristics of a device, its location, functions (services) and the processes by which interactions with other *domoDevices* must take place. Through *DomoML*, data type models are also standardized to provide a suitable intermediate representation for conversion to and from inbound and outbound values and thereby enable data marshalling between heterogeneous technologies (Figure 3).

- *domoMessage* provides a formal way to describe an event, a command or a response. A message can belong to different types: *command*, when it requests execution of a service belonging to a *domoDevice; success*, when the service is successfully executed; *failure*, when the request for the service has not been correctly executed; *event*, when there is a *domoDevice* status change (Figure 4).

Once this process of defining and describing single functions (*profile* definition) has been completed, the next step is to associate these control information units with corresponding active control elements, which are identified as services.

Since these services all map to the same *domoML* language, they can be considered equivalent as far as interoperability is concerned. They define and constitute a new control layer, which can be seen as a meta-infrastructure of high-level services. This logical infrastructure binds several single domotic systems, providing them with common information exchange and control mechanisms.

The implementation of device and service discovery is different for each *TechManager*, since it is strictly dependent on the underlying technology. At the time of system configuration, *DomoNet* assigns to each device detected by the *TechManagers* a unique *id* and the *URL* of its managing Web service, so that it can be addressed from within and outside the framework.

It should be noted that these technology choices are enabling factors for remote control, as well as interoperability. In fact, *domoNetWS* is an actual Internet node designed to share environments and services in a distributed fashion with any other *domoNetWS* or *DomoNet* client application in order to monitor and control all home environments at a distance through the use of Internet-capable equipment such as PCs, PDAs, Tablets, Smartphones and so on, irrespective of the technologies adopted in the specific domotic devices, but using *domoML* alone. In fact, by following the publish/subscribe policy, all *DomoNet* client applications can receive notifications sent by *DomoNet* regarding state changes in any domotic device and send the appropriate commands to be executed.

By way of example, Figure 3 shows a *DomoML* description of two devices that are to be made interoperable. The first is a *living room lamp* managed by *KNX* domotic technology. It offers two services: *getStatus* and *setStatus*, both of which take the *boolean* value *ON/OFF* (the former as output, the second as input). When the *setStatus* service is invoked, the system evaluates the *linkedService* tag, which indicates that the *setPower* service of the device with *id* = “*3*” is associated to the *setStatus*. The *id* = “*3*” refers to the *UPnP*-technology *radio* device, defined in the second device in the figure, with tag *device description* = “*Radio*”. The *setStatus* service has the internal variable *status*, whose value, assigned at the service call invocation, is copied into the input variable *power* of the *UPnP Radio* device's *setPower* service. The actual association is defined via the tag *linkedInput* of the *setStatus* service of the *living room lamp* device, thereby also achieving sharing of the state (*ON* or *OFF*) between devices belonging to different domotic technologies.

Figure 4 illustrates a message used to execute a command activating a device. The service invoked is *setStatus* of the device *id* = “*1*” (for example the *living room light* in the previous example), which sets the value of its input variable *status* to *ON*. The message will then be read and interpreted by the relevant *TechManager*, which translates it into the specific language of the device's domotic technology. The *TechManager* then sees to forwarding the message to the command target device, according to the rules and format of the specific domotics system. To communicate the outcome of the command to be executed, the system produces a feedback message of either success or failure. In the event that the user manually activates the device, the relevant *TechManager* recognizes the event and consequently produces and broadcasts a message identical to the previous one, but with the *message* tag attribute *messageType* set to the value *UPDATE*. Then *domoNetWS* identifies it and finds the properly configured device and service in order to execute the corresponding action (e.g., turn the light on).

This modular structure is designed to ensure that future developments may include more protocols within the interoperability framework.

The *DomoNet* framework enables building fully interoperable domotic systems and provides a way to abstract information on the domotic environment to implement AmI principles. It is the basic infrastructure that allows building “intelligent” applications operating at a level above it, amongst which a system that learns users' habits and behaviors in order to anticipate their needs.

## 4. Meeting User Needs

In our everyday lives we usually carry out the same, often related, actions to achieve certain goals. By way of example, when we leave home, we usually switch off all the appliances, close all the windows, activate the alarm system and so on. With the passing of time, these actions become habits and are often performed at specific times of the day or are related to certain events. By monitoring users' activities in a highly enriched domotic home environment, it is possible to learn user habits and anticipate user needs. A software system able to analyze data provided by sensors can take decisions and act in the users' stead.

In pervasive environments, context is defined as the knowledge that a system has about its own state. Such knowledge takes the form of a state representation, though this definition is not exhaustive due to the constraints intrinsic in detection technologies and the logic limits of context management algorithms. In any event, context information is a valuable resource for pervasive applications, since it allows for gathering information about the real world and thereby adapting applications in a consistent manner. Context is not simply a snapshot of the pervasive environment [28] at a particular time, but instead usually represents information over a given period of time during the life of the environment itself. It provides the knowledge bases for systems that learn from past contexts and experiences; it provides advanced adaptive capacities and facilitates proactive decision support with different degrees of autonomy.

**Figure 5.**
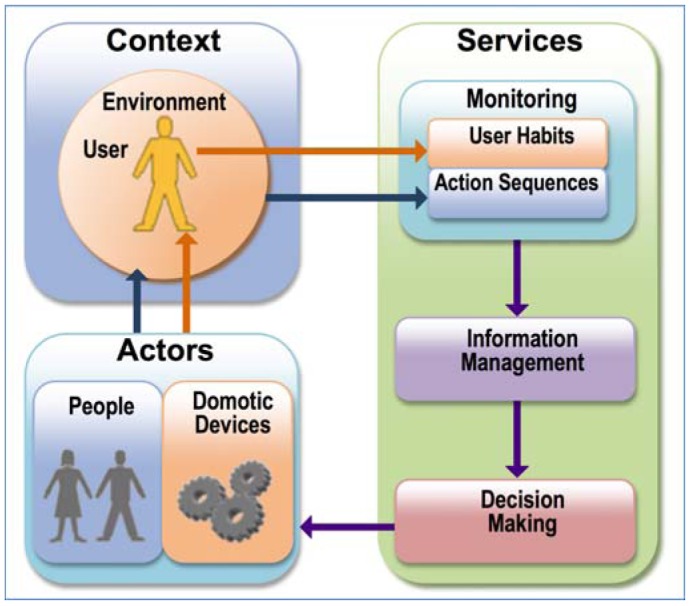
Scheme of AmI based systems.

As shown in Figure 5, the first step in the life cycle of an *agent-programmed* rules system is the acquisition of information about the users and their environment by means of a monitoring service software module. The data is then analyzed and processed by the information manager module. A decision-making software application then uses this processed information to identify the actions to be performed using machine learning techniques. Lastly, the *decision maker's* requests are translated into commands and sent to the recipient domotic devices, which, together with any actions on the part of occupants, modify the initial setting.

Such advanced Ambient Intelligent scenarios must be based on the principles underlying *Artificial Intelligence*. Using an *Artificial Intelligence* system based on an *agent-programmed* rules paradigm, the system configuration stage is limited to the physical installation of the devices and software without regard for adjustments and settings, which often can be difficult to understand and put into practice. The users can just continue to live inside their living space as usual and just ignore the technology surrounding them.

### 4.1. The Intelligent Prototype

Exploiting the approach outlined above, isolated devices must be integrated in order to achieve global, unified goals. *DomoNet* has been applied to tackling this problem. By virtue of its capacity to abstract the peculiarities of underlying heterogeneous, currently mainstream, well-established domotic technologies, it enables them to co-exist and interwork, without eliminating their mutual differences.

As shown in Figure 2 the AmI prototype *DomoPredict* [29] is a component of the *DomoNet* project, implemented, like all *DomoNet* clients, as an independent software client able to send commands and receive notifications of any state change in any domotic device through the *DomoNet* framework and the exchange of *domoMessages*.

*DomoPredict* behaves as an independent intelligent entity that makes decisions autonomously and responds to *DomoNet* by sending commands to manage any device conforming to any domotic technology wherever it is found (Figure 2). Such characteristics make *DomoPredict* a true software agent that runs continuously and makes decisions on its own, as it is capable of perceiving the context, coming to a decision and reacting to such perceived situations appropriately, all without human intervention.

**Figure 6.**
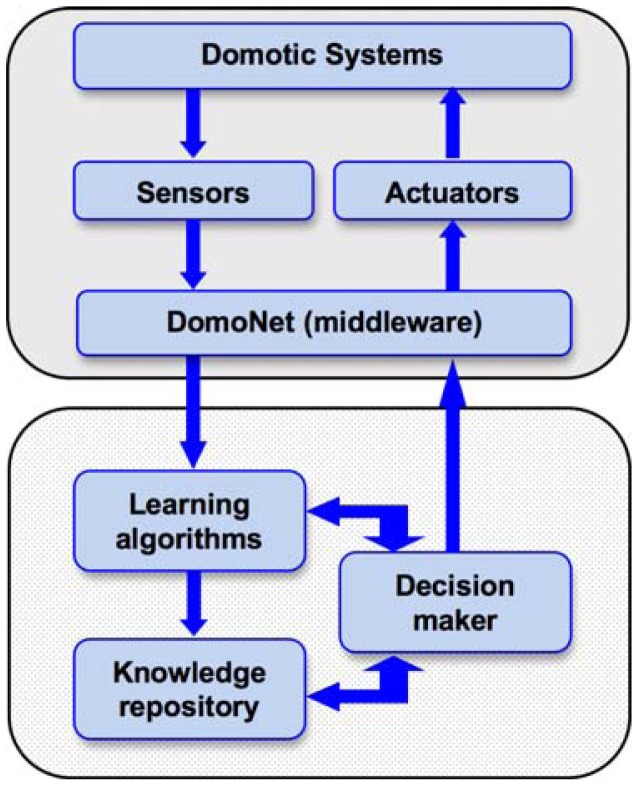
The DomoPredict architecture.

In particular (Figure 6), our AmI solution is based on the use of a set of sensors and actuators. Sensor output data are collected and analyzed by the *DomoNet middleware*, which reorganizes and uniformizes the information coming from the different domotic systems. *Learning algorithms* make a comparison between new input data and the *knowledge repository*. The *learning algorithms'* computations can enhance and improve the information stored in the *knowledge repository*. The *Decision maker* exploits the information stored in the *knowledge repository* and the data from the middleware in order to arrive at a ‘diagnosis’, and to automatically act, if needed, using the system *actuators*.

Actuation of the learned rules is executed via a command message sent to the *DomoNet* middleware server side, which routes it to the appropriate devices. In order to optimize results, the solution includes two complementary, interoperable modules that perform real-time analyses to identify user behaviors and to verify whether previously established rules are still valid. The two modules are:

1. *Association rules manager*: this module applies a *Data Mining* paradigm to carry out the analyses necessary to learn scenarios from device usage data. The literature contains many other machine-learning approaches, such as *neural networks* or some variant of *Bayesan* classifiers or *Markov* models. The choice of the *Data Mining* approach, however, offers a number of advantages: it facilitates the management of a non-ordered list of actions; requires lower computational times; and enables nontrivial extraction of implicit, previously unknown, but potentially useful information from data [30].
  The algorithm learns action sequences that constitute user habits. An action sequence is a set of actions that occurs within a fixed short time span or that is executed with systematic periodicity (*i.e.*, each day at the same time). These action sequences are learned using the *unsupervised learning method* of the *association rules*. This method enables producing rules as implications between binary partitions of the representing the learned scenarios, in the form {X => Y}, where X and Y are disjunct itemsets, so as to anticipate subsequent actions the moment the user carries out the preceding ones. The frequent itemsets corresponding to the potential scenarios are generated by the *Apriori* algorithm, which selects candidates through the method *F(k* − *1)* × *F(k* − *1)* [31].
  The strength of an *associative rule* can be measured as a function of its *support* and its *confidence. Support* represents the fraction of transactions that contain both X and Y, while *confidence* instead represents how often the elements in Y are also present in a transaction containing X. The problem of discovering *associative rules*, given a set of transactions, consists in finding all the rules whose *support* and *confidence* values are equal to or greater than preset thresholds.
  The science of extracting useful information from large data sets or databases currently faces a number of challenges [32]. In fact, the approach followed poses such problems as obtaining efficient summaries of large amounts of data and identifying the structures and links within a data set in order to construct predictors for future events.
  The size of the dataset is moreover very important for the proper functioning of data mining algorithms. Indeed, data sets can be very large, often requiring days of computer time to create a single model. From this prospective, usual data mining is unsuitable for our purposes, because the dataset is empty at startup and is created in real time via the updates broadcast messages sent by *DomoNet* when a device state changes. The solution found to this obstacle is to act on the *support* parameter of the *Apriori* algorithm, bearing in mind that the few data available initially could lead to the acquisition of erroneous habits. In order to limit such erroneous habit acquisition, the dataset is enriched with a new itemset only when the minimum *support* parameter is greater than a prefixed threshold.
  As this parameter is used to evaluate whether or not a group of actions is frequent, simply increasing its value when dealing with small datasets will make it more difficult for a given scenario to be learned, thereby preventing infrequent itemsets from being considered. Thus, at first, a rather high value is set, which is then decreased in the long term proportionally with the increasing population size of the dataset, so that eventually most itemsets will be deemed frequent, thereby allowing even rather rare habits to be learned.
  One essential requirement for the software's efficiency is the ability to quickly adapt to users' habits. This goal is achieved by implementing a *reinforcement* function in the machine learning procedure. This function is activated by users unconsciously when they correct undesired or incorrect system actions. For example, if the system enters a scenario that calls for switching on a light, and the user switches off that very light, this fact permits the system to ‘understand’ that this particular scenario is incorrect. The erroneous rules are relegated to a blacklist and cannot be re-learned for a period of time proportional to the number of times the user has provided negative feedback.
2. *Statistical rules manager*: the second module is designed to learn the parameters of scenarios that are not captured by the *Data Mining* approach, for instance, the user's living quarter preferences, such as temperature and lighting levels for a favorite room, or scenarios related to the identification of anomalies that do not comply with the preference rules.
  To this end, the module creates a user profile obtained by statistically analyzing the frequency and percentage of appliance usage. This data is obtained by monitoring *DomoNet* activities according to the preferences learned from experience. This statistical inference is calculated at preset intervals over the course of an entire day.
  Collected data are recorded in structures called *UsageTables* in the pair format <*device state*, *percentage*>, which indicate, over the timeframe in question, either the percentage time a device is in a particular state, or the percentage time that the user has performed some action, for instance, listening to his/her favorite music or maintaining a certain room temperature. A number of different timeframes (daily, weekly, seasonal and perpetual) have been considered and a different *UsageTable* created for each. Such data are used to satisfy user preferences through a conditional rule of the sort *condition* => *set preferences*, where the condition is dictated by specific events, such as for example, waking up, returning home, a new season's start, and so forth.

### 4.2. The Work Cycle Steps

The lifecycle is activated every time a device belonging to the system changes its state and consists of execution of the following steps in sequence:

1. *Collection of information regarding the use of devices*: When a device changes its state, an update multicast message is sent from *DomoNet* to all clients that have subscribed to the update notification, according to the *publish/subscribe* design pattern paradigm. In this way *DomoPredict* analyzes the update message and uses it to build and enhance the rules dataset. At the same time, the *log manager* archives the information about the updates in an *XML* file for diagnostic purposes and future analyses.
2. *Analysis of the collected information for creating rules*: This is done by observing the relations between actions and time, adopting *unsupervised* machine learning techniques. Inhabitant preferences are determined by statistically analyzing the percentage use of devices as stored in a database of events generated by the household devices themselves.
3. *Creation/removal of rules based on analysis results*: The algorithm checks whether the set of actions is performed every day within the same time interval. If so, the relative scenario will therefore be created and activated each day automatically at a certain hour and minute, as determined by calculating the mean time at which it is enacted on previous days. Otherwise, a scenario will be created that automatically identifies the minimum set of actions that enables identifying subsequent ones. This last aspect is the most important and critical feature of the system and can be better explained with an example.
  Let us suppose that the system has learned a scenario that includes the two rules: “switch on the light in the living room” and “switch on the TV”. Once the user has switched on the living room light, it must be determined whether (s)he wants to turn on the TV as well. To do this, it is necessary to calculate the probability that the performance of a group of actions belonging to a scenario imply execution of the others in that same scenario. The conditional relation need not be one of certainty—a high probability is sufficient grounds for anticipating the need.
  Such a procedure necessarily requires a stage of preprocessing of the data, so as to determine the minimum set of actions that enables recognizing the correct scenario. To obtain useful results, we must first arrive at some groupings of potentially correlated actions, which will be represented by those performed in succession over a brief time interval. Such time interval is a system parameter, called the “*correlation window*”, whose duration can be adjusted according to need. It is worthwhile underlining that the efficiency of the algorithm does not depend on the order of the completed actions, but only on the temporal correlations existing between the actions performed, since the actions making up a scenario are not always performed in the same order. This implies that the *Apriori* algorithm refers to a set of unordered items (actions). Moreover, acquired scenarios are continually subject to modification.
  The learning of scenarios through the analysis of the users' preferences is fundamental to fulfilling all the system's potentialities. Indeed, not all scenarios can be learned by simply observing the actions that users habitually perform within their living environment. For instance, observation alone cannot convey to the system either the temperature or musical preferences of each single user. A statistical solution has been implemented to infer such information by estimating the preferences of each user registered in the smart environment. Such preferences are learned over time by analyzing certain parameters measured during users' daily usage of the domotic devices. The system is set up to monitor and learn: temperature, lighting, favorite musical genre and the vital functions of the different users, so as to enable constant monitoring of their state of health. In the event that anomalous values are detected, the system can estimate the hazard level and act accordingly by performing specific pre-established actions, which may range from making simple adjustments to increase an ill user's comfort, to initiating emergency calls (to the physician, family members, *etc.*) in more serious situations.
  The rules learned by the system can consequently change over time with varying user habits or when particular events or changes due to external factors occur. In such circumstances, the system is able to remove and eventually substitute previously learned rules that are no longer valid due to the newly acquired experience.
4. *Rules execution*: This is activated when a pre-established action time is reached or when a user enters the environment and is recognized by the system, or in relation to recognition of the minimum expected set of actions.

## 5. System Validation

A working prototype has been developed according to the architecture described above and tested at the *Domotics Laboratory* of the *ISTI-CNR* (Italian National Research Council) in Pisa. It was successfully deployed mounted on mobile panels at a stable demo center during an Ambient Assisted Living project. It has also been demonstrated at important technology exhibitions and used as a reference for didactic purposes by various universities. Some very impressive features, usually not feasible in such systems, have been achieved by exploiting a combination of domotic technologies.

The implementation features five *TechManagers* related to five home domotic systems (*UPnP*, *Konnex*, *MyHome*, *X10* and *BacNet*). *UPnP* is used to control multimedia devices in order to manage audio and video content; *Konnex*, *MyHome*, *X10* and *BacNet* are used to manage home appliances and systems.

All *Automatic Learning* systems require a rather large training database for correct validation. In our case, the implementation choices dictate that the dataset be compiled in real time, starting from scratch. This choice offers the immediate advantage of enabling the scenarios to be learned from the start. Learning scenarios through the *Data Mining* technique known as associative rule learning would require a sufficiently extensive dataset [33]. To avoid the risk of the system acquiring incorrect habits due to the limited initial size of the dataset, at the beginning a low *Apriori* support parameter is maintained in such a way that the dataset is sufficiently full in a short time. The best results were obtained by setting the initial *Apriori* threshold support and confidence parameters at 10% and 90%, respectively. Therefore, by limiting the analysis to the latest period alone, we were able to obtain sound results, despite the lack of a large body of historical data.

**Figure 7.**
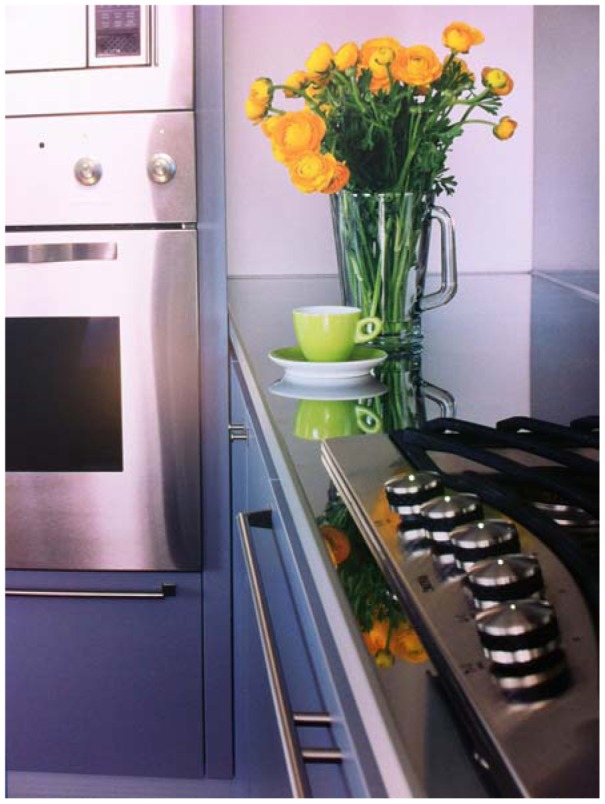
The CNR Domotics Lab Demo Center.

Despite the limited size of the dataset, we have thus been able to carry out an accurate evaluation of the system's learning capacity. System validation tests have been conducted by monitoring the activities of four volunteer colleagues in the domotic environment of our laboratory. Each volunteer subject underwent testing for one week (Figure 7). Although all subjects had the same assignments, performing the test with four different subjects ensured that they would be carried out in different ways and through different sequences. This avoided the risk of unreliable test results based on a sole individual consistently performing the same actions in the same sequence, and thereby enabled an important test of the software system's capacity to deal with variability.

The volunteers' assignments involved carrying out customary daily habits under usual circumstances, that is, to simply perform a series of repetitive activities within the test setting. Each habit was formulated without indications as to having to carry out the activities at the same time or in the same sequence. During testing the subjects were allowed to interact with any device; however, any two successive actions had to be carried out within a prefixed timeframe, set at 15 seconds. Habits had to be performed in relation to precise circumstances, which were: “waking up in the morning and having breakfast”, “going out”, “returning home from work in the evening and having supper”, and “after supper until going to bed”.

During the test all interactions were collected through the devices; the average rate was 42.8 actions each day. In order to reduce system training time and subjects' length of stay in the laboratory, before performing this field test system usage data, represented by simulated habits, were collected for the equivalent duration of a further four weeks by random data generation with information replication. In particular, we represented believable day-to-day activities, which were interspersed with 100 pseudo-random actions per day in order to make the data more realistic. We thus accumulated available data on eight weeks of system use.

Verification of proper learning of the rules was performed based on acceptance of the system action in question by the volunteer. If, within a preset time, the subject voluntarily intervened by activating at least one device among those making up part of some rule, it meant that that rule had not been learned correctly.

At the end of the test, system validation was carried out according to the *K-Fold Cross Validation* method. The data have been divided into eight sets, each corresponding to one week of data collection. The data were then used in alternation for the learning stage and simulated tests.

The resulting data made up the knowledge base for *DomoPredict*. Various experimental tests of the application were then performed, modifying the following system variable parameters from time to time:

- the *size of the timeframe* used to establish whether the same action has been performed habitually over the most recent period and the length of the analysis period itself;
- the initial *support*, *confidence* level, *correlation time* interval (used to determine whether two consecutive actions are potentially related) and the *reinforcement time* interval (used to establish whether an action performed after a system command is sent represents potential reinforcement to the learning);
- the *size of the time interval* used to aggregate data, the percentage threshold to establish whether a given device state is normal and the analysis period.

This methodology has enabled dividing the results according to four different types of actions and identifying for each the parameter values that yield the best results:

1. *First action type*: composed of a set of actions usually carried out by the user, but unrelated to the time of performance. The best results were obtained by setting the size of the time window to fifteen minutes and the analysis period to one week; with such values, the validation performed using the available dataset yielded a specificity value of 89%, 88% sensitivity and 89% accuracy;
2. *Second action type*: made up of one or more actions usually executed at the same time of day. The best results were obtained with a correlation time interval of 45s and reinforcement time interval of 30 s; with such values the validation performed on the available dataset yielded a specificity value of 82%, 88% sensitivity and 85% accuracy;
3. *Third action type*: created by a set of actions that permits the system to configure the user environment using his/her personal preferences. The validation was performed separately for the different scenario aspects to be learned:
  - preferred temperature yielded an accuracy of 86%;
  - musical tastes yielded an accuracy of 79%;
  - lighting intensity of the different dimmer-equipped lamps in different areas yielded an accuracy of 92%.
4. *Fourth action type*: learns the user's preferences along a time scale by analyzing the state of the system at precise moments during the day; such preferences are then activated at a set time. Combination of the optimal values calls for an aggregation time interval of 15 m, a threshold percentage of 80% and an analysis period of one week; using these values for validation yielded a specificity value of 85%, 88% sensitivity and 87% accuracy.

The rules produced by *DomoPredict* during execution of the test are:

- “At 7:30 am each day => set thermostat to 21 °C, switch on bedside lamp, open bedroom blinds, switch on bathroom light”;
- Successful activation of fingerprint reader => switch on living room light, switch on dining room light, switch on kitchen light, switch on kitchen TV, deactivate intruder alarm, set thermostat to 21 °C;
- Detection of occupant in living room => switch on TV;
- At 11:00 pm each day => close all blinds and switch off all lights, close water and gas electromagnetic valves, activate intruder alarm, set thermostat to 18 °C;
- Preferred temperatures 11:00 pm–07:30 am => 18°;
- Preferred temperatures 07:30 am–11:00 pm => 21°;
- Preferred music type upon reentry => Jazz;
- Preferred music type on awakening => Classical.

## 6. Conclusions

Apart from making life more comfortable for users without particular needs, the software system can offer significant advantages to the elderly and/or disabled people as well. For these segments of the population, even the simplest of everyday actions may represent an insurmountable obstacle, hence a system that learns their habits and performs actions in their stead can offer much needed support and safeguards. It can moreover contribute to reducing the currently acute problem of the digital divide. Using this system can also provide advantages in terms of energy savings. Once the system has acquired and learned good practices such as switching off lights and closing shutters depending on the time of day or in relation to a change in season, this information will never be lost and subsequent automatic system actions will be able to contribute significantly to saving energy.

The results obtained and reported in the foregoing serve to illustrate the effectiveness of the approach adopted and its potential for use in cooperation with all existing domotic systems without the use of specialized software for each type of device. The implementation of the automatic learning system is able to learn types of habits, which enable covering a large part of the common behaviors of Ambient Intelligence system users, and can anticipate such users' behaviors quite reliably.

With regard to related work following similar approaches, none seems able to cover all the aspects considered by *DomoPredict* in anticipating user needs. Solutions based exclusively on statistical algorithms, such as Tapia [16], can estimate only general, non-specific user needs, because they do not consider individual actions as sequences, but only customary user habits over rather long time periods. Such an approach is however especially useful for monitoring user behaviors to detect any deviations or abnormities. Instead, approaches based on data mining techniques alone, such as Lühr [17], cannot formulate an overview of a given user's general habits, because they focus solely on recognizing specific sequences of actions.

The choice of following a hybrid approach–applying both the *Data Mining* technique of associative rule learning and statistical learning methods–has rendered the system more versatile and reliable. In fact, combining the two forms of learning has led to a summing of the strong points of the two different learning methods, while limiting their respective shortcomings.

The system developed fits well within the perspective and goals of current *Ambient Intelligence* research. The methods applied for learning the behaviors and habits of *AmI* system users enables the system to anticipate their needs quite well. Moreover, system performance improves over time, as new experience is accumulated. The number of errors committed by the system is relatively low right from the start and then falls further as the system acquires ulterior data.

The prototype clearly requires more thorough, longer-duration testing, in order to improve learning and enable more careful evaluation of the system parameters. A more extensive dataset will surely enable more accurate validation and evaluation of the system's capabilities. The *DomoPredict* system thus represents a good point of departure for future development, with the main goal of improving the learning capacity achieved so far.

## 7. Future Work

The next important step is to enhance the system's robustness. The system has not yet been tested with a large number of devices, and a more robust approach to information storage (e.g., device descriptions, mapping tables, *etc.*) should be followed using appropriate tools, such as a relational database instead of the current approach of file dumping. After this crucial migration, it will be possible to focus on the security of the framework using two main techniques: classical authentication and access control to database records, and communication protection procedures such as message coding, especially between remote *DomoNet* instances.

Future framework versions will most likely move towards a more comprehensive semantic approach [34] in the definition of devices and services, allowing for greater context awareness and user friendliness. These *Ambient Intelligence* features will be achieved by investigating the use of *ontologies* and the *Semantic Web* to provide *DomoPredict* with semantic tools to enable it to exploit knowledge representation of the environment at a much higher level of abstraction and to increase the usability of the *domoML* abstraction layer. The most immediate benefit of this will be to enable the system to understand user needs and then act with a higher degree of autonomy. For example, it will be possible to assign the system a specific goal without specifying all the actions needed to achieve it, so activation of the necessary devices may vary from time to time depending on user and environmental conditions. In particular, this calls for the use of dedicated ontologies able to account for every possible type of device in a home (e.g., lamps, multimedia players and so on), as well as all such devices' various functions (e.g., power on, play and so on), including their impact within the environment and all the consequent logical associations between device, events, impacts, *etc.* The *ontology* will also have to provide for descriptions of the various rooms in a home and their furnishings and include spatial concepts such as “near”, “up”, “down” and so on. This will be the basis for future creation of a friendly human-machine interface (*HMI*) based on the use of natural language to enable the use of utterances such as “The lamp on the table” or more simply, “More light in the bedroom” in order to achieve the desired effects. In addition, in future releases of our system, the agent paradigm can be applied not only to implementing *AmI* systems, but rather to all stages of the software development life cycle.

The results obtained from development of the *DomoPredict* framework can be applied to the medical field, specifically to anticipate health problems [35]. In fact, a system that automatically acquires user habits can infer a particular state of illness or discomfort by comparing the latest user action sequence with previously collected data. Such development represents a great challenge. Today's major telecare systems only come into play in the event of an emergency, which is often too late. Thus, being able to anticipate and recognize certain behaviors or events heralding serious health problems in time can often save lives.

Further important future work will be dedicated to enabling the system to identify specific users and their locations, for instance when they enter or leave a room. To improve the identification of inhabitants, an *RFID*-based strategy [36] can be employed to enable the system to recognize who performs an action, when and where. Although *RFID* technology has been traditionally used only for objects in fields such as logistics supply chains and inventory management, there are many other applications where it is necessary to identify, not only things, but also people in order to be able to properly manage the context. Clearly, such future trends will go a long way to realizing the ideal of human environments replete with “hidden technology” serving people in an unobtrusive fashion.

## Figures and Tables

**Table 1.** Main Domotic Alliances and Working Groups.

**Standard**	**Media**	**Description**
**Bluetooth**	RF	Technology specification for small form factor, low-cost, short range radio links between mobile PCs, mobile phones and other portable devices.
**CEBus**	All	Developed by the Electronic Industries Association (EIA) to support the interconnection and interoperation of consumer products in a home.
**EIA 570A**	Twisted	Residential Telecommunications Cabling Standard
**HAVI** (Home Audio Visual Interoperability)	IEEE	Consumer Electronics (CE) industry standard that will ensure interoperability between digital audio and video devices from different vendors.
**HBS** (Home Bus System)	Coax Twisted Pair	A consortium of Japanese companies, supported by government agencies and trade associations, encompasses links among appliances, telephones, and audio-video.
Home Plug and Play	All	Provides interoperability among products with multiple transport protocols. Overseen by the CEBus Industry Council.
HomePlug Alliance	Power Line	The alliance's objective is to enable and promote rapid availability and adoption of cost effective, interoperable and specification-based home power line networks and products.
IEEE 802.15.4	Wireless	A low data rate solution with multi-month to multi-year battery life and very low complexity. It is intended to operate in an unlicensed, international frequency band.
**JINI**	All	It provides simple mechanisms which enable devices to plug together to form an impromptu community without any planning, installation, or human intervention.
**KNX**	All	Link sensors and actuators to building systems that control HVAC, security, access, and life safety. The common association of EIB, BatiBUS and HES.
**LonMark** Interoperability Association	All	The LonMark Association's mission is to enable the easy integration of multi-vendor systems based on LonWorks networks using standard tools and components.
**OSGI** (Open Service Gateway Initiative)	All	To create an open standard for a service gateway that is inserted between the external network and the internal network.
**UPnP** (Universal Plug and Play)	All	Industry group of companies promoting Universal Plug and Play networking protocols and device interoperability standards.
**ZigBee**	Wireless	Association of companies working together to enable reliable, cost-effective, low-power, wirelessly networked, monitoring and control products based on an open global standard.
